# Niche differentiation in nitrogen metabolism among methanotrophs within an operational taxonomic unit

**DOI:** 10.1186/1471-2180-14-83

**Published:** 2014-04-04

**Authors:** Sven Hoefman, David van der Ha, Nico Boon, Peter Vandamme, Paul De Vos, Kim Heylen

**Affiliations:** 1 Laboratory of Microbiology, Department of Biochemistry and Microbiology, Ghent University, Ghent, Belgium; 2 Laboratory of Microbial Ecology and Technology (LabMET), Ghent University, Coupure Links 653, B-9000 Ghent, Belgium; 3 BCCM/LMG Bacteria Collection, Ghent, Belgium

**Keywords:** *Methylomonas methanica*, *Methylomonas koyamae*, *Methylomonas lenta*, Strain dependency, Nitrogen assimilation, Detoxification

## Abstract

**Background:**

The currently accepted thesis on nitrogenous fertilizer additions on methane oxidation activity assumes niche partitioning among methanotrophic species, with activity responses to changes in nitrogen content being dependent on the *in situ* methanotrophic community structure Unfortunately, widely applied tools for microbial community assessment only have a limited phylogenetic resolution mostly restricted to genus level diversity, and not to species level as often mistakenly assumed. As a consequence, intragenus or intraspecies metabolic versatility in nitrogen metabolism was never evaluated nor considered among methanotrophic bacteria as a source of differential responses of methane oxidation to nitrogen amendments.

**Results:**

We demonstrated that fourteen genotypically different *Methylomonas* strains, thus distinct below the level at which most techniques assign operational taxonomic units (OTU), show a versatile physiology in their nitrogen metabolism. Differential responses, even among strains with identical 16S rRNA or *pmoA* gene sequences, were observed for production of nitrite and nitrous oxide from nitrate or ammonium, nitrogen fixation and tolerance to high levels of ammonium, nitrate, and hydroxylamine. Overall, reduction of nitrate to nitrite, nitrogen fixation, higher tolerance to ammonium than nitrate and tolerance and assimilation of nitrite were general features.

**Conclusions:**

Differential responses among closely related methanotrophic strains to overcome inhibition and toxicity from high nitrogen loads and assimilation of various nitrogen sources yield competitive fitness advantages to individual methane-oxidizing bacteria. Our observations proved that community structure at the deepest phylogenetic resolution potentially influences *in situ* functioning.

## Background

Methane is a major greenhouse gas, accounting for up to 20-30% of global warming effect [1]. Aerobic methanotrophic bacteria constitute the main microbial methane sink through their ability to derive energy from its oxidation to carbon dioxide using the key enzyme methane monooxygenase [2]. Cultured representatives of this functional guild are phylogenetically positioned within the *Gammaproteobacteria* (Type I methanotrophs, forming two clades, i.e. Type Ia and Ib), the *Alphaproteobacteria* (Type II methanotrophs) [3,4], and the *Verrucomicrobia*[5]. These methanotrophs appear physiologically diverse, especially in regard to their nitrogen metabolism [6], with strains demonstrating co-metabolic oxidization of ammonia [4], nitrate reduction to nitrite [7], detoxification of hydroxylamine and nitrite to the greenhouse gas nitrous oxide via a multitude of pathways [8-10], and fixation of atmospheric nitrogen [11-14].

The effect of nitrogen, under the form of either ammonium- or nitrate-based fertilizers, on methane fluxes in soils is a widely studied topic of global concern. Unfortunately, available reports are not in agreement, with methanotrophic activity after nitrogen addition either showing no effect, inhibition (by non-specific ionic effects [15], competitive inhibition between methane and ammonia or the formation of toxic intermediates [10,16]) or stimulation (by relief of nitrogen limitation), resulting in identical or altered associated community composition [17-20]. The currently accepted thesis assumes niche partitioning among methanotrophic species, with methane oxidation activity responses to changes in nitrogen content being dependent on the *in situ* methanotrophic community structure [19,21].

Unfortunately, widely applied tools for microbial community assessment, based on short read 16S rRNA gene sequencing techniques or proxies thereof (such as denaturating gradient gel electrophoresis (DGGE) or terminal-restriction fragment length polymorphism (T-RFLP)), only have a limited phylogenetic resolution mostly restricted to genus level diversity [22], and not to species level as often mistakenly assumed. As a consequence, intragenus or intraspecies metabolic versatility in nitrogen metabolism was never evaluated nor considered among methanotrophic bacteria as a source of differential responses of methane oxidation to nitrogen amendments. Nevertheless, we know from other organisms that ecophysiology can be strain-specific, and closely related bacteria can occupy distinct niches [23,24]. This was for example elaborately demonstrated in *Prochlorococcus*, with cultured strains having distinct pigmentation, maximum growth rates, metal tolerances, nutrient utilizations and photophysiological characteristics [23]. Also, among methanotrophic genera of the same type (I or II), ammonia co-metabolisation and product inhibition was found to be organism-specific, although in this study it is difficult to ascertain the taxonomic level at which differences occur as only two strains from different genera were tested for each type [10].

We hypothesized that strains within the same methanotrophic genus, thus below the level at which most techniques assign operational taxonomic units (OTU), can demonstrate a large physiological versatility in their nitrogen metabolism. If so, this would make current microbial community structure analyses less suitable for evaluating changes in *in situ* methanotrophic communities after for example fertilization. To this end, we explored the differential response of various aspects of the nitrogen metabolism within fourteen genotypically distinct members of the genus *Methylomonas* (Type Ia), including the species *M. methanica* and *M. koyamae*. Their tolerance to high ammonium, nitrate, nitrite and hydroxylamine concentrations, their ability to fix nitrogen, and their capacity to produce nitrite and nitrous oxide from nitrate or ammonium were qualitatively evaluated. *Methylomonas* is a relevant genus for such a comparison, since *Methylomonas* strains are ubiquitous in nature [4,7,25-27] and nitrogenous fertilization was reported to stimulate some of its members in rice field soils [19]. Two non-*Methylomonas* strains belonging to Type Ib and Type II were included as references.

## Results

### N_2_ fixation

We screened a strain set of fourteen genotypically distinct *Methylomonas* strains, one Type Ib strain (*Methylococcaceae* sp. R-49797) and one Type II strain (*Methylosinus* sp. R-45379) in batch experiments for their ability to fix dinitrogen gas as sole nitrogen source. Only at low oxygen tension, ten out of the thirteen *Methylomonas* strains could grow without any nitrogen source except for N_2_ in the headspace (Table 1), confirming the presence of an oxygen sensitive nitrogenase. Since oxygen became rapidly limiting in the batch setup used, volumes of air equal to the initial volume were injected (up to eight times) to allow further activity and growth of the cultures. Maximum OD_600nm_ values averaged at 0.332 ± 0.122 (start OD_600nm_ values of 0.017 ± 0.019) across strains, while activity was observed with drops in methane and oxygen levels and subsequent increase in CO_2_ levels (data not shown). No growth or activity was observed for the strains cultivated in parallel in nitrogen-free medium under high oxygen tension, confirming the absence of nitrogenous compounds in the growth medium, which was also demonstrated by colorimetric analysis of nitrate, nitrite and ammonium. To confirm observations, the presence of the *nifH* gene encoding the Fe protein of nitrogenase was verified. All strains that did not give a *nifH* gene amplicon were also negative for growth and activity in nitrogen-free medium at high and low oxygen tension.

**Table 1:** Nitrogen assimilation and tolerance per strain

	***Methylomonas methanica *** **strains**							***Methylomonas koyamae *** **strains**					***Methylomonas lenta***		**Type Ib**	**Type II**
	**NCIMB 11130**^**T**^	**R-45362**	**R-45363**	**R-45364**	**R-45371**	**R-45372**	**R-45374**	**NCIMB 14606**^**T**^	**R-45378**	**R-45383**	**R-49799**	**R-49807**	**R-45370**	**R-45377**	**R-49797**	**R-45379**
NaCl (+NO_3_^−^)	100 mM	100 mM	100 mM	40 mM	100 mM	100 mM	100 mM	100 mM	100 mM	100 mM	100 mM	100 mM	200 mM	200 mM	100 mM	40 mM
NaCl (+NH_4_^+^)	40 mM	100 mM	100 mM	40 mM	100 mM	40 mM	100 mM	100 mM	100 mM	100 mM	150 mM	100 mM	200 mM	200 mM	100 mM	40 mM
NaNO_3_ & KNO_3_^a^	40 mM	40 mM	40 mM	40 mM	40 mM	40 mM	40 mM	40 mM	40 mM	40 mM	40 mM	40 mM	100 mM	100 mM	40 mM	20 mM
NH_4_Cl^b^	40 mM	40 mM	40 mM	40 mM	40 mM	40 mM	40 mM	100 mM	40 mM	40 mM	100 mM	40 mM	40 mM	40 mM	100 mM	40 mM
(NH_4_)_2_SO_4_-N^b^	40 mM	40 mM	40 mM	40 mM	40 mM	40 mM	40 mM	40-100 mM^c^	40 mM	40 mM	100 mM	40 mM	40 mM	40 mM	100 mM	40 mM
NaNO_2_	2 mM	2 mM	2 mM	2 mM	2 mM	2 mM	2 mM	2 mM	2 mM	2 mM	2 mM	2 mM	2 mM	2 mM	5 mM	-
Hydroxylamine^d^	1 mM	-	1 mM	1 mM	1 mM	1 mM	-	1 mM	-	-	1 mM	-	-	-	-	1 mM
*nifH* presence	+	+	+	+	+	+	+	+	+	+	+	+	+	+	-	+
N_2_ fixation^e^	+	+	+	+	+	+	+	+	+	+	+	-	-	-	-	+

Values represent maximum concentration supporting growth of the culture (n = 2).

^a^For nitrate as sole nitrogen source, 2, 10, 20, 40, 150 and 200 mM with NaNO_3_ and KNO_3_ were tested. Due to a technical error, strains R-49799, R-49807, R-45370, R-45377, R-49797 and R-45379 were not tested with KNO_3_ concentrations above 10 mM.

^b^For ammonium as sole nitrogen source, 2, 10, 20, 40, 150 and 200 mM NH_4_Cl and (NH_4_)_2_SO_4_-N were tested.

^c^Different outcome for duplicates;

^d^Hydroxylamine tolerance was evaluated in dAMS supplemented with 0.01, 1 and 2 mM hydroxylamine;

^e^Under low O_2_ tension only: +, positive and -, negative for growth based on OD_600nm_ values in nitrogen-free medium.

Three tested *Methylomonas* strains (R-49807, R-45370 and R-45377) were not active and did not grow in nitrogen-free medium under low oxygen tension during the 21-days experiment, although PCR and sequence analyses demonstrated the presence of a *nifH* gene. *M. koyamae* R-49807 unexpectedly did not exhibit N_2_ fixation, although this strain shares a 100% 16S rRNA and a >99% *nifH* sequence similarity with strains NCIMB 14606^T^ and R-49799 (data not shown), both positive for N_2_ fixation under low oxygen tension. *M. lenta* R-45370 and R-45377 typically grew slower than the other strains (one to two-week lag phase in liquid media under optimal conditions). Therefore, it is possible that these strains would fix N_2_ after longer incubation periods.

### Assimilation of nitrate and ammonium and tolerance to high levels

Assimilation of and/or tolerance to high levels of ammonium and nitrate was evaluated, in addition to the influence of general osmotic effects tested with equimolar concentrations of sodium chloride (Table 1). These tests were qualitatively scored based on growth, which was used as a proxy for methane oxidation as both features were shown to correlate (Additional file 1: Figure S1). This experiment was performed in microtiter plates, in which all strains could grow well, even though a more limited gas transfer is expected in such a setup compared with batch experiments due to a lack of mixing. The complete strain panel was able to use both ammonium and nitrate for assimilation and strains could cope in general with a maximum of 40 mM amended nitrogen, except the type II strain, which only tolerated a maximum of 20 mM NO_3_^−^-N (Table 1).

Two *M. koyamae* strains, R-49799 and NCIMB 14606^T^, showed a higher tolerance for NH_4_^+^-N, up to 100 mM. Notably, growth at these high ammonium concentrations was not supported for *M. koyamae* R-49807, showing gene sequence similarity with *M. koyamae* NCIMB 14606^T^ and *M. koyamae* R-49799 of 100% for the 16S rRNA gene (Additional file 2: Figure S4, Additional file 3: Table S1) and between 97-100% for the *pmoA* gene encoding the particulate methane monooxygenase, the key enzyme for methane oxidation (Additional file 4: Figure S5, Additional file 5: Table S2). In addition, out of these three strains only R-49799 exhibited higher sodium chloride tolerance when grown with ammonium as sole nitrogen source over nitrate (150 mM compared with 100 mM). For *M. methanica* cultures, strains NCIMB 11130^T^ and R-45372 exhibited higher salt tolerance when cultivated with NH_4_^+^-N instead of NO_3_^−^-N. *M. methanica* R-45364 was salt sensitive regardless of the nitrogen source and demonstrated an equal tolerance to sodium chloride, ammonium and nitrate (≤40 mM). The other *M. methanica* strains tolerated higher sodium chloride concentrations than equimolar nitrate and ammonium concentrations, most likely due to compound-specific stress [15]. Only *M. lenta* strains R-45370 and R-45377 grew in the presence of 100 mM nitrate and tolerated higher concentrations of nitrate than ammonium (maximum of 40 mM), whilst also showing the highest salt tolerance regardless of their nitrogen source (200 mM NaCl). The other strains tolerated higher sodium chloride additions than equimolar nitrate additions, again suggesting concentration-dependent nitrate inhibition.

### Assimilation and toxicity of the potential intermediates nitrite and hydroxylamine

Ammonium amendments introduce a fraction of ammonia to the culture depending on the pH (NH_3_/NH_4_^+^; pKa = 9.23). Ammonia can be toxic to methanotrophs as it functions as a competitive inhibitor of MMO or leads to accumulation of hydroxylamine and nitrite, toxic products of its oxidation. Tolerance of methanotrophs to ammonia can thus be dependent of intrinsic toxicity levels of hydroxylamine and nitrite as well as their ability to use either of them for assimilation. This was again tested qualitatively in microtiter plates using growth as a proxy for methane oxidation.

For our strain panel, preliminary experiments showed that none of the strains could grow with 1 mM or 2 mM hydroxylamine as sole nitrogen source. Therefore, tolerance of hydroxylamine was tested by providing 2 mM ammonium for assimilation and spiking of 0.01 mM, 1 mM and 2 mM hydroxylamine. None of the strains could grow in the presence of 2 mM hydroxylamine. Eight strains exhibited growth when 0.01 mM and 1 mM hydroxylamine was added, while eight other strains did not even show growth at a concentration of only 0.01 mM hydroxylamine (Table 1).

All *Methylomonas* strains were able to grow with up to 2 mM nitrite as sole nitrogen source, demonstrating nitrite tolerance as well as assimilation. The type Ib strain R-49797 tolerated the highest nitrite concentration (up to 5 mM). However, this strain was sensitive to hydroxylamine, even at concentrations of 0.01 mM. Type II strain could not use nitrite as sole nitrogen source.

### Production of nitrite and nitrous oxide from nitrate and ammonium

**Figure 1:**
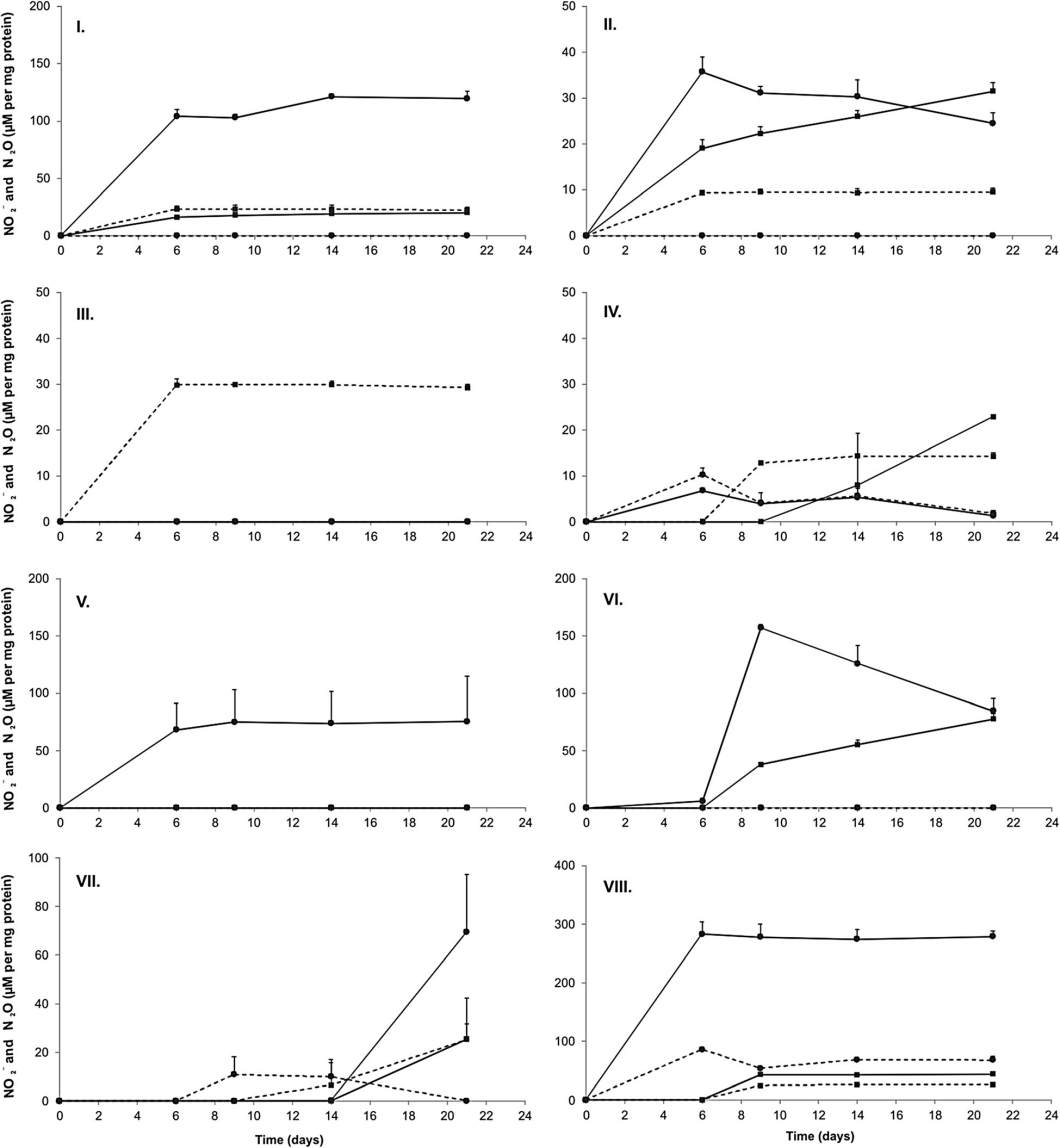
**Specific nitrite and nitrous oxide production profiles for assigned phenotypes.** Nitrite (circles) and nitrous oxide (squares) production of methanotrophic strains cultivated in nitrate medium (dNMS, solid line) and ammonium medium (dAMS, dashed line). Nitrous oxide values showed are for total vial and were corrected to N_2_O-N to allow equimolar nitrogen comparison with NO_2_^−^. The Y-axis could not be made uniform for all strains for clarity; care should be taken when interpreting the data between strains. Phenotype I is represented by *M. methanica* NCIMB 11130^T^, phenotype II by *M. koyamae* R-45378, phenotype III by *M. methanica* R-45363, phenotype IV by *M. methanica* R-45371, phenotype V by *M. koyamae* R-45383, phenotype VI by *M. koyamae* R-49807, phenotype VII by *M. lenta* R-45370, Phenotype VIII by *Methylosinus* sp. R-45379.

To evaluate nitrite and nitrous oxide production from nitrate or ammonium, strains were grown in batch cultures at an initially high oxygen tension (O_2_ levels in air). Because of methanotrophic activity, oxygen concentrations gradually decreased to levels too low to support further methane oxidation, thus inducing stationary growth phase. Most strains clearly produced nitrite (15 out of 16) and nitrous oxide (13 out of 16) from nitrate, while production of nitrous oxide, but not nitrite, from ammonium was also a general feature (14 out of 16 strains). Nitrite production from ammonium was only apparent for *Methylosinus* sp. R-45379 (Type II) but near detection limit (10 μM) for *M. methanica* R-45371 and *M. lenta* R-45370 and R-45377. The strain panel could be divided into eight different dissimilatory nitrogen phenotypes, based on detection of nitrite, nitrous oxide or both (Table 2); onset of N-species production or amount produced was not taken into account. An example of each phenotype is displayed in Figure 1. It should be noted that monitoring only started at late exponential phase (day 6), as preliminary experiments then showed the onset of nitrous oxide production. Therefore, influence of nitrate or ammonium on actual methane oxidation rate was not assessed, although small amounts of methane were still consumed (Additional file 6: Figure S2) and final methane concentration consumed with either N source did not differ per strain.

**Table 2:** Production of nitrite and nitrous oxide from nitrate or ammonium per strain (n = 2)

		**dNMS (10 mM KNO**_**3**_**) cultivation**			**dAMS (10 mM NH**_**4**_**Cl) cultivation**		**Phenotype**
		**NO**_**2**_^**− **^**production**	**N**_**2**_**O production**	**NO**_**2**_^**−**^**↘ & N**_**2**_**O↗**^**a**^	**NO**_**2**_^**− **^**production**	**N**_**2**_**O production**	
*M. methanica*	NCIMB 11130^T^	+	+	-	-	+	I
	R-45362	+	+	+	-	+	II
	R-45363	-	-	-	-	+	III
	R-45364	+	+	-	-	+	I
	R-45371	+/−^b^	+	-	+/−^b^	+	IV
	R-45372	+	+	+	-	+	II
	R-45374	+	+	+	-	+	II
*M. koyamae*	NCIMB 14606^T^	+	+	+	-	+	II
	R-45378	+	+	+	-	+	II
	R-45383	+	-	-	-	-	V
	R-49799	+	+	+	-	+	II
	R-49807	+	+	+	-	-	VI
*M. lenta*	R-45370	+	+	Nt^c^	+/−^b^	+	VII
	R-45377	+	Nt^c^	Nt^c^	+/−^b^	+	VII
Type Ib	R-49797	+	+	+	-	+	II
Type II	R-45379	+	+	-	+	+	VIII

^a^Observed decline in NO_2_^−^ levels and corresponding rise in N_2_O levels over time.

^b^+, positive result (>10 μM NO_2_^−^ or N_2_O-N); −, negative result (<10 μM NO_2_^−^ or N_2_O-N); +/−, Values around the detection limit of the assay (10 μM);

^c^Nt, Not tested due to slow growth of the strain.

Detectable amounts of nitrite were produced from nitrate, used as sole nitrogen source, in all strains once oxygen concentrations became low. The only exception was *M. methanica* R-45363 (Table 2; Figure 1: phenotype III). When growth and methane oxidation activity ceased due to oxygen limitation, no additional nitrite was produced, but several strains (Figure 1: phenotype II and VI) showed a slow decrease in nitrite levels with a corresponding rise in nitrous oxide over time (Table 2). This was most clear for *M. koyamae* R-49807, as for this strain from the start of the incubation at high oxygen tension (O_2_ levels in air) until day six (approximately 1.7% v/v O_2_), methane was oxidized but no nitrite or nitrous oxide could be measured. At day nine, nitrite levels of approximately 400 μM were measured at oxygen levels below 0.3% v/v, and from that point onwards, until the end of the incubation at day 21, nitrite levels dropped with a corresponding rise in nitrous oxide levels, while no more methane oxidation occurred. *M. koyamae* R-45383 produced nitrite but did not produce nitrous oxide (Table 2; Figure 1: phenotype V). *M. methanica* NCIMB 11130^T^ and R-45364 and *Methylosinus* sp. R-45379 produced both nitrite and nitrous oxide, but did not show a subsequent drop in nitrite and corresponding rise in nitrous oxide (Table 2; Figure 1: phenotypes I, IV and VIII). *M. methanica* R-45371 produced nitrous oxide and small amounts of nitrite, approximating the detection limit of the assay (10 μM).

With ammonium as sole nitrogen source, *M. methanica* R-45371, *M. lenta* R-45370 and R-45377, and *Methylosinus* sp. R-45379 (Table 2; Figure 1: phenotypes IV, VII and VIII respectively) produced both measurable levels of nitrite and nitrous oxide. Interestingly, between day six and nine, a fraction of the produced nitrite seems to be converted into nitrous oxide, after which both nitrite and nitrous oxide levels remained stable over time [Table 2; Figure 1: phenotypes IV and VIII (phenotype VII deviates because of slow growth)]. Most strains were able to produce nitrous oxide without preceding measureable levels of nitrite.

## Discussion

The ability of *Methylomonas* strains to produce nitrite and nitrous oxide from moderate levels (10 mM) of nitrate and ammonium, indicative of detoxification, demonstrated metabolic variability on both genus- and species level (Table 2; Figure 1). Ammonium amendments introduce a fraction of ammonia to the culture depending on the pH, which can be oxidized to hydroxylamine by methane monooxygenase. The conversion of hydroxylamine directly to nitric oxide or indirectly via nitrite is well studied and limited to the activity of hydroxylamine oxidoreductase [8-10], and to a much lesser extent cytochrome P460 [6]. In contrast, nitrate metabolism is underexplored in methanotrophs and little is known, except that nitrite from nitrate can be produced by both assimilatory and dissimilatory nitrate reductases [6,28]. Nevertheless, high levels of nitrite produced from nitrate should also be converted to nitric oxide for nitrite detoxification. Upon production, the cytotoxic nitric oxide needs to be immediately detoxified to the, at least for the cell, harmless nitrous oxide by one of the many known nitric oxide reductase enzymes [29-33]. In the present study, most strains were able to produce nitrous oxide from ammonium without preceding measurable levels of nitrite, either due to an immediate conversion of hydroxylamine to nitric oxide by hydroxylamine oxidoreductase or to small transient nitrite peaks below detection levels. The latter hypothesis is plausible when considering the nearly identical nitrous oxide profiles observed for *M. methanica* NCIMB 11130^T^ and *Methylosinus* sp. R-45379, except the nitrite peak (Figure 1: phenotypes I and VIII), suggesting the same detoxification mechanism. Nitrate as sole nitrogen source was converted to nitrite in all strains once oxygen concentrations became low, except for *M. methanica* R-45363 that probably lacks an enzyme for nitrate reduction. Strains of both *M. methanica* and *M. koyamae* showed a drop in nitrite levels with a corresponding rise in nitrous oxide levels (Table 2; Figure 1). These observations would fit nicely with activities of an oxygen-sensitive nitrate reductase producing nitrite in actively growing cells, which is subsequently detoxified to nitrous oxide (via nitric oxide, not measured in this study) during stationary phase, similar to previous observations for non-denitrifiers [34,35], but unreported in methanotrophs. *M. methanica* NCIMB 11130^T^ and R-45364 produced both nitrite and nitrous oxide from nitrate, but did not show a subsequent drop in nitrite and corresponding rise in nitrous oxide (Table 2; Figure 1: phenotypes I, IV and VIII). *M. koyamae* R-45383 was the only strain not able to produce nitrous oxide from either nitrate or ammonium, probably because it lacks a nitric oxide reductase. On the other hand, *M. koyamae* strain R-49807 did produce nitrous oxide from nitrate but not from ammonium. This indicates that the nitrous oxide produced was truly derived directly from nitrate via nitrite, and not via an indirect process via ammonium, i.e. assimilatory reduction of nitrate to ammonium. It is clear that, although variable phenotypes for nitrite and nitrous oxide production from nitrate and ammonium can be tentatively explained using the currently described genomic inventories of other methanotrophs [6] or a combination thereof, various novel features on specifically nitrate reduction should be the subject of further genome and expression studies.

Within a single species, strain-dependent differences were also observed. This was most obvious for strains within *M. koyamae* regarding their tolerance to high ammonium levels. Noteworthy are the identical 16S rRNA gene and (almost) identical functional gene sequences between the highly ammonium-tolerant *M. koyamae* strains NCIMB 14606^T^ and R-49799 (up to 100 mM) and the low ammonium-tolerant strain R-49807 (up to 40 mM) (Additional file 2: Figure S4 and Additional file 4: Figure S5; Additional file 3: Table S1 and Additional file 5: Table S2). This differentiation among *M. koyamae* strains is further demonstrated in their tolerance to hydroxylamine (up to 1 mM). In addition, R-49807 unexpectedly did not exhibit N_2_ fixation, as sole *M. koyamae* strain, despite a >99% *nifH* gene sequence similarity with strains NCIMB 14606^T^ and R-49799, both positive for N_2_ fixation under low oxygen tension. This might be explained by a higher oxygen sensitivity of the nitrogenase of this strain [14,36], requiring oxygen concentrations lower than the 2% used here. In addition, something worth considering as well is the applicability of *M. koyamae* R-49799 for methane mitigation in high-ammonium sites, since this strain grows at 100 mM ammonium levels, utilizes 2 mM nitrite as sole nitrogen source, tolerates hydroxylamine levels up to 1 mM and possesses pathways to detoxify ammonia and nitrite.

Some features appeared to be general within *Methylomonas* with some strain specific exceptions. The observed higher tolerance to ammonium than nitrate confirmed previous reports of methanotrophic growth inhibition above 40 mM nitrate [37,38]. Nevertheless, two *M. lenta* strains (R-45370 and R-45377) tolerated nitrate concentrations up to 100 mM. This could not be linked to a possible higher nitrite tolerance, as all strains could grow with up to 2 mM nitrite as sole nitrogen source. This nitrite tolerance and assimilation was in contrast to earlier findings that nitrite utilization was rare for *M. methanica* members [7]. The presence of the *nifH* gene and the ability to fix nitrogen with an oxygen-sensitive nitrogenase was also found in most strains, although both traits appear to be strain-dependent when combining the results of this study with other reports [12]. Although initially thought to be limited to mostly Type II and Type Ib methanotrophs [11], our findings are in agreement with more recent reports suggesting that nitrogen fixation is a common feature of many methanotrophs, including Type Ia and verrucomicrobial methanotrophs [12-14].

## Conclusions

Microbial ecologists still struggle to link microbial community structure to ecosystem functioning, notwithstanding that in-depth analyses of microbial community structure has never been easier than now, with the affordability of deep sequencing of amplified genes as well as whole communities. But to make sense out of sequence data, basic knowledge about the biology of the microorganisms involved in the biogeochemical functions under study is pivotal. Unfortunately, current insights are often retrieved from sporadic pure culture studies including only few distantly related strains. Here, investigation of closely related, genotypically different methanotrophic strains revealed differential responses to overcome inhibition and toxicity from high nitrogen loads and assimilation of various nitrogen sources, yielding competitive fitness advantages to individual methane-oxidizing bacteria. We have not assessed the specific effect of nitrogen on methanotrophic activity rate, but rather demonstrated the surprising versatility below the commonly used cut-off for operational taxonomic units, i.e. genus level. Our results proved that community structure at the deepest phylogenetic resolution potentially influences *in situ* functioning. Until molecular tools become available to allow much finer analyses of microbial diversity, metabolic variability at those unmeasurable levels should be taken into account.

## Methods

### Bacterial strains and standard growth conditions

Fourteen of the sixteen tested strains were members of the genus *Methylomonas*: the type strain *Methylomonas methanica* NCIMB 11130^T^ and six strains R-45362, R-45363, R-45364, R-45371, R-45372 and R-45374 most closely related to this type strain (98.3-98.6% 16S rRNA sequence similarity; further referred to as *M. methanica* strains); the type strain *Methylomonas koyamae* NCIMB 14606^T^ and four strains R-45378, R-45383, R-49799 and R-49807 most closely related to this type strain (97.9-100% 16S rRNA sequence similarity; further referred to as *M. koyamae* strains); two strains *Methylomonas* sp. R-45370 and R-45377 most closely related to the no-longer extant type strain of *M. scandinavica* (97.5% 16S rRNA sequence similarity) and recently described as novel species *Methylomonas lenta*[39]. All *Methylomonas* strains were genetically different as determined by GTG_5_ rep-PCR fingerprinting [40]. In addition, two non-*Methylomonas* strains were included as a reference: *Methylococcaceae* sp. R-49797, a member of the *Methylococcus*-*Methylocaldum*-*Methylogaea* clade (Type Ib) and *Methylosinus* sp. R-45379 (Type II). Included strains were obtained from various origins: strains R-45362, R-45363, R-45364, R-45371, R-45372, R-45374 and R-45370 were isolated from the top layer of a denitrification tank of a waste water treatment plant, strain R-45377 was isolated from a slurry pit of a cow stable; strains R-45378, R-45383 and R-45379 were isolated from a wetland [40]; strains R-49807 and R-49797 were isolated from a facultative waste stabilization pond in South Africa. Strain R-49799 was isolated from a high ammonium (70 mM) methanotrophic enrichment from a mixture of inocula (compost heap, top soil with leaf litter, anaerobic sludge and a wastewater treatment plant). All strains were routinely subcultured on diluted nitrate mineral salts (dNMS) plates [41] and incubated in gastight jars (Oxoid, UK) under a CH_4_ : air (1 : 1) atmosphere. The composition of dNMS in this study was the following: 2 mM KNO_3_, 4 mM MgSO_4_, 0.9 mM CaCl_2_, 2 mM Na_2_HPO_4_/KH_2_PO_4_ buffer (pH 6.8), 14 μM ferric-sodium-EDTA and a trace element solution [25] with Cu^2+^ concentration adjusted to 10 μM. Fresh colonies from one-week grown cultures on dNMS plates were used as start inoculum (OD_600nm_ 0.01, final concentration) for the different growth experiments performed in this study. To be able to reach comparable cell densities, two-week grown cultures were used for the two slower-growing strains *M. lenta* R-45370 and R-45377.

### Assessment of nitrogen assimilation and toxicity

The ability of the strains to grow in the presence of a range of concentrations of NaNO_3_, KNO_3_, NH_4_Cl and (NH_4_)_2_SO_4_-N (2, 10, 20, 40, 150 and 200 mM) and NaNO_2_ (2, 5, 10 mM) was assessed. In order to evaluate general osmotic influences, sodium chloride tolerance was also tested in dNMS and dAMS (identical medium composition with ammonium instead of nitrate as sole nitrogen source), each supplemented with 40, 100, 150 and 200 mM NaCl. Hydroxylamine tolerance was evaluated in dAMS supplemented with 0.01, 1 and 2% (w/v) hydroxylamine. Initial cultivation experiments showed that strains cultivated with ammonium consistently preferred a 10 mM buffer over a 2 mM buffer, while the opposite was observed with nitrate as nitrogen source. Therefore, in this study, dNMS (nitrate, nitrite or nitrogen-free medium, see below) media contained a 2 mM phosphate buffer, while dAMS (ammonium) media contained a 10 mM phosphate buffer. Besides nitrogen source and buffer concentration, all other components were identical between media.

All strains were inoculated in duplicate in liquid media in sterile 96-well microtiter plates (300 μL, final volume per well). Cultures were incubated under a CH_4_ : air (1 : 1) atmosphere at optimal temperature (20°C for strains R-45370, R-45371 and R-45377 and 28°C for the other strains). Growth was monitored and verified by visual inspection after 4, 7, 11, 14 and 21 days of incubation.

### Assessment of production of nitrite and nitrous oxide

Initial experiments on a limited number of *Methylomonas* strains showed that nitrous oxide was produced in cultures grown with ammonium or nitrate as sole nitrogen source once a stationary phase was induced via oxygen depletion, while nitrogen was not depleted. Nitrous oxide production was never detected in nitrite-containing media that were not inoculated or in cultures with nitrite levels too high to support growth and activity, ruling out nitrous oxide production from nitrite from a non-biological origin. Therefore, all strains were cultivated in dAMS with 10 mM NH_4_Cl and in dNMS with 10 mM KNO_3_ (a high enough nitrogen concentration to prevent nitrogen depletion in our batch setup) in gastight serum vials under a 20% CH_4_ in air atmosphere and time points were selected to include only the stationary phase (expected to start before or around day six, except for several slower growing cultures such as *Methylomonas lenta* R-45370 and R-45377), namely after 6, 9, 14 and 21 days of incubation. Growth, methane oxidation activity, either nitrate or ammonium consumption, and nitrite and nitrous oxide production were assessed. In this batch setup, oxygen was the growth-limiting factor, and thus the setup does not allow to distinguish between effects caused by cells entering the stationary phase or by oxygen limitation.

### Analytical methods

Methane oxidation activity was assessed by monitoring of CH_4_, O_2_ and CO_2_ levels via gas chromatography using a Compact GC (Global Analyzer Solutions, Belgium) equipped with two columns (O_2_/N_2_ and CO_2_/N_2_O separation) connected to a thermal conductivity detector and one column (CH_4_ levels) connected to a flame ionization detector. The change in gas pressure due to methane oxidation was monitored with an Infield 7 pressure meter (UMS, Germany). Values measured by gas chromatography were converted to μmol gas L_liquid_^−1^ by compensating for change in gas pressure and taking the solubility of the gases into account. Colorimetric assays were performed to determine the nitrite [42], nitrate [43] and ammonium [44] concentration. Growth was monitored via OD_600nm_ and, when necessary, converted to mg protein via the correlation factor 243.77 obtained from previous growth experiments [45] (Additional file 7: Figure S3). For miniaturized screening set up, growth was scored positive when the average OD_600nm_ value became larger than the average initial OD_600nm_ value added by 5-times its standard deviation. Growth correlated very well with methane oxidation (Additional file 1: Figure S1) and was therefore used as a proxy thereof.

### Nitrogen fixation

Strains were inoculated in duplicate as explained above in liquid nitrogen-free dNMS in gastight flasks under a 20% CH_4_ in air atmosphere (approximately 21% O_2_, high oxygen tension condition) and under a 20% CH_4_ in ten-fold diluted air (with helium) atmosphere (approximately 2.1% O_2_, low oxygen tension condition). In addition to the Type Ib strain for which *nifH* gene amplification was negative, four extra methanotrophic strains without the *nifH* gene were included as negative controls, which indeed all did not demonstrate growth because of inability to fix dinitrogen. Growth was determined through OD_600nm_ measurements and verified by visual inspection after 4, 7, 11, 14 and 21 days of incubation. In addition, the methane oxidation activity was assessed by gas chromatography. Absence of nitrate (below 0.15 mM), ammonium (below 0.15 mM) and nitrite (below 10 μM) in nitrogen-free dNMS was confirmed via colorimetry.

### *pmoA and nifH* gene sequence analyses

DNA was extracted by alkaline lysis [46]. Amplification of *pmoA* gene encoding the particulate methane monooxygenase, the key enzyme for methane oxidation, was performed using the primer set A189f/mb661r as described previously [47]. Amplification of the *nifH* gene encoding the highly conserved Fe protein of nitrogenase was performed using the primer set F1/nifH439R, the PCR mix and temperature program as described by De Meyer et al. [48]. When amplification was positive, gene sequences were generated and sequences were assembled with the BioNumerics 5.1 software (Applied Maths, Belgium). Protein translation analysis using Transeq (http://www.ebi.ac.uk/tools/emboss/transeq) and pBLAST [49] confirmed the genes encoding functions.

### Nucleotide sequence accession numbers

The *nifH* gene sequence data generated in this study have been deposited in GenBank/EMBL/DDBJ with accession numbers HF954360, HF954362 and HF954366 to HF954376. The *pmoA* gene sequence data generated in this study have been deposited in GenBank/EMBL/DDBJ with accession numbers HF954359, HF954361, HF954363 and from HG915717 to HG915727.

## Competing interests

The authors declare to have no competing interests.

## Authors’ contributions

SH and KH conceived and designed experiment, interpreted data and wrote the manuscript. SH acquired and analysed data. DvdH, NB, PV and PdV critically revised the manuscript. All authors read and approved the final manuscript.

## Supplementary Material

Additional file 1: Figure S1 Correlation between growth (OD_600nm_) and methane consumption. In general, growth correlates well with methane oxidation **(A)**, validating the use of growth as a proxy for methane oxidation (n = 8). However, the correlation between growth and methane oxidation is influenced by growth rate and growth medium. When taken this into account for a single strain, both parameters correlate even better, as exemplified for R-45374 in dNMS **(B)**. Click here for file

Additional file 2: Figure S4 16S rRNA gene phylogenetic neighbor-joining tree. The publically available 16S rRNA gene sequences of the strains included in this study were aligned with type strains of the remaining species of the genus *Methylomonas* using the integrated aligner of ARB [50]. Based on the 16S rRNA gene alignment, a neighbor joining tree was constructed using the MEGA 5 software [50]. The maximum composite likelihood method was used and 1186 positions were taken into account. Bootstrap analysis based on 500 replicates was performed. Bootstrap values below 70% are not shown. Bar: 0.02 substitutions per nucleotide position. Click here for file

Additional file 3: Table S1 Estimates of Evolutionary Divergence between 16S rRNA gene sequences. The number of base substitutions per site between 16S rRNA gene sequences are shown. Pairwise distance analyses were conducted based on the alignment used in Additional file 4: Figure S4. Evolutionary analyses were conducted in MEGA5 [51]. Click here for file

Additional file 4: Figure S5 *pmoA* gene phylogenetic neighbor-joining tree. The *pmoA* gene sequences of the strains included in this study and available *pmoA* gene sequences of type strains of the remaining species of the genus *Methylomonas* were aligned based on translated protein sequences and DNA-based neighbor joining trees constructed using MEGA5 [51]. The maximum composite likelihood method was used and 396 positions were taken into account. Bootstrap analysis based on 500 replicates was performed. Bootstrap values below 70% are not shown. Bar: 0.05 substitutions per nucleotide position. Click here for file

Additional file 5: Table S2 Estimates of Evolutionary Divergence between *pmoA* gene sequences. The number of base substitutions per site between *pmoA* gene sequences are shown. Pairwise distance analyses were conducted based on the alignment used in Additional file 4: Figure S5. Evolutionary analyses were conducted in MEGA5 [51]. Click here for file

Additional file 6: Figure S2 Methane consumption in late exponential to stationary phase in dNMS **(A)** or dAMS **(B)** per strain (n = 2). Assigned phenotypes are indicated between brackets. Error bars are high for R-45370 because of slow growth of one replicate. Click here for file

Additional file 7: Figure S3 Correlation between growth (OD_600nm_) and protein content. Data was taken from [45]. A correlation factor was extracted for conversion of growth to protein content. Click here for file
