# Supplementary figures and images for: Niche differentiation in nitrogen metabolism among methanotrophs within an operational taxonomic unit
Source: BMC Microbiol. 2014 Apr 4;14:83. doi: 10.1186/1471-2180-14-83 (PMC3997834; doi:10.1186/1471-2180-14-83)

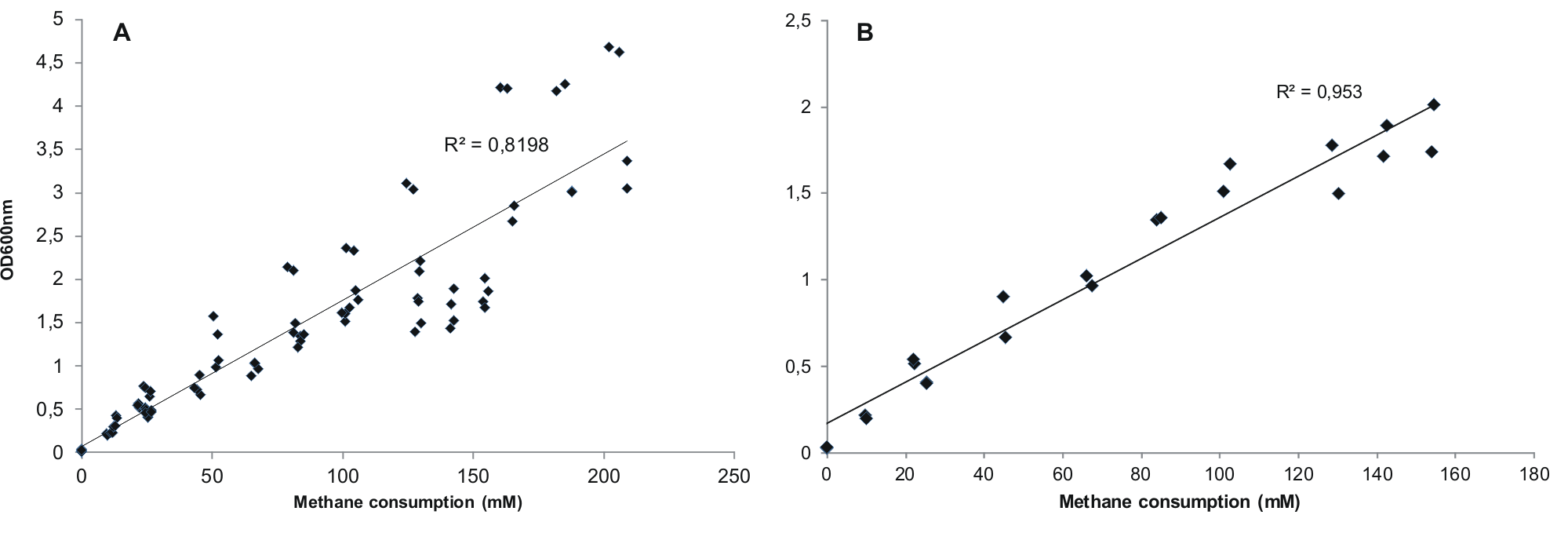

Supplement: Additional file 1: Figure S1 — Correlation between growth (OD600nm) and methane consumption. In general, growth correlates well with methane oxidation (A), validating the use of growth as a proxy for methane oxidation (n = 8). However, the correlation between growth and methane oxidation is influenced by growth rate and growth medium. When taken this into account for a single strain, both parameters correlate even better, as exemplified for R-45374 in dNMS (B). [file 1471-2180-14-83-S1.tiff]

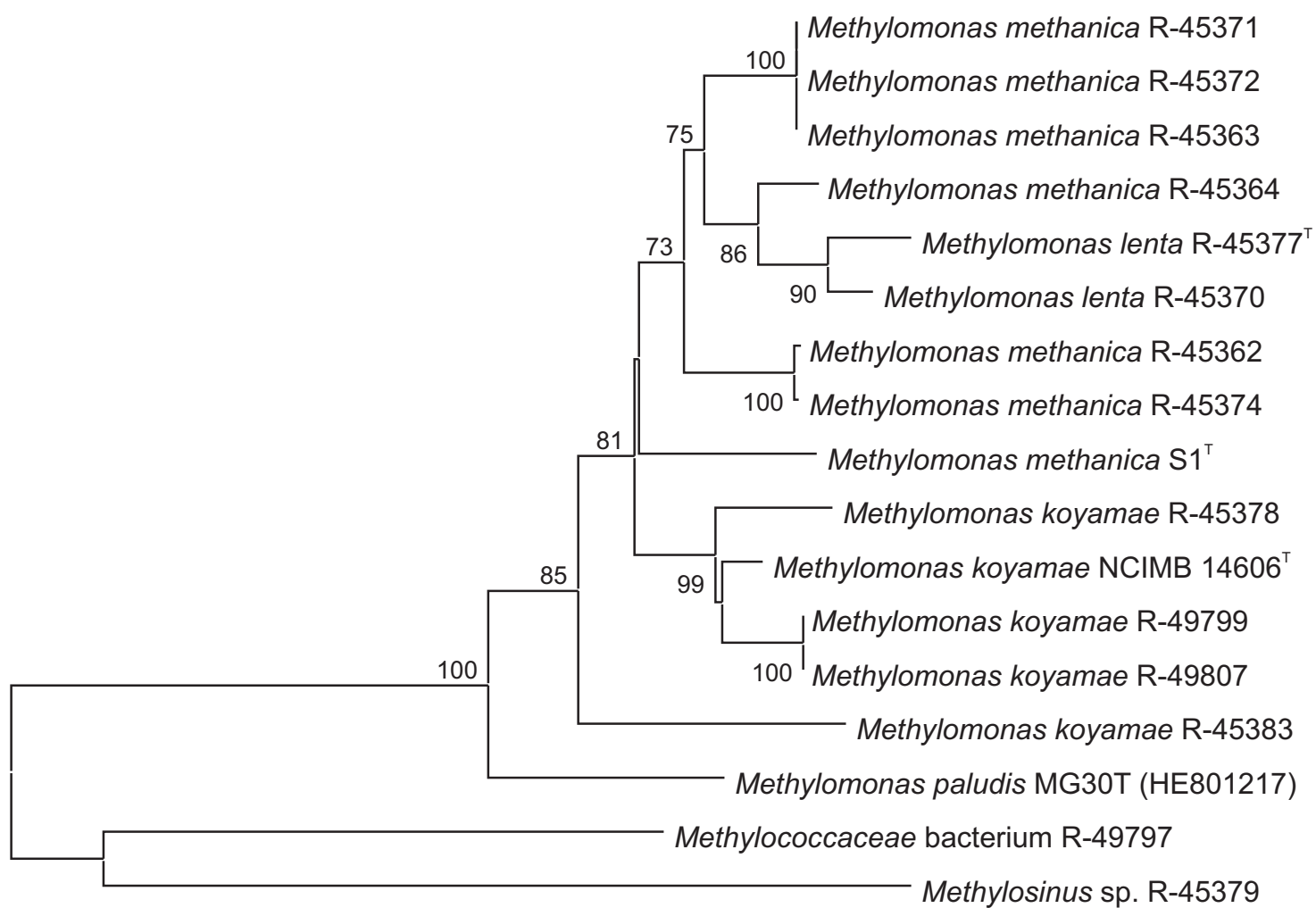

0,05

Supplement: Additional file 4: Figure S5 — pmoA gene phylogenetic neighbor-joining tree. The pmoA gene sequences of the strains included in this study and available pmoA gene sequences of type strains of the remaining species of the genus Methylomonas were aligned based on translated protein sequences and DNA-based neighbor joining trees constructed using MEGA5 [51]. The maximum composite likelihood method was used and 396 positions were taken into account. Bootstrap analysis based on 500 replicates was performed. Bootstrap values below 70% are not shown. Bar: 0.05 substitutions per nucleotide position. [file 1471-2180-14-83-S4.pdf]

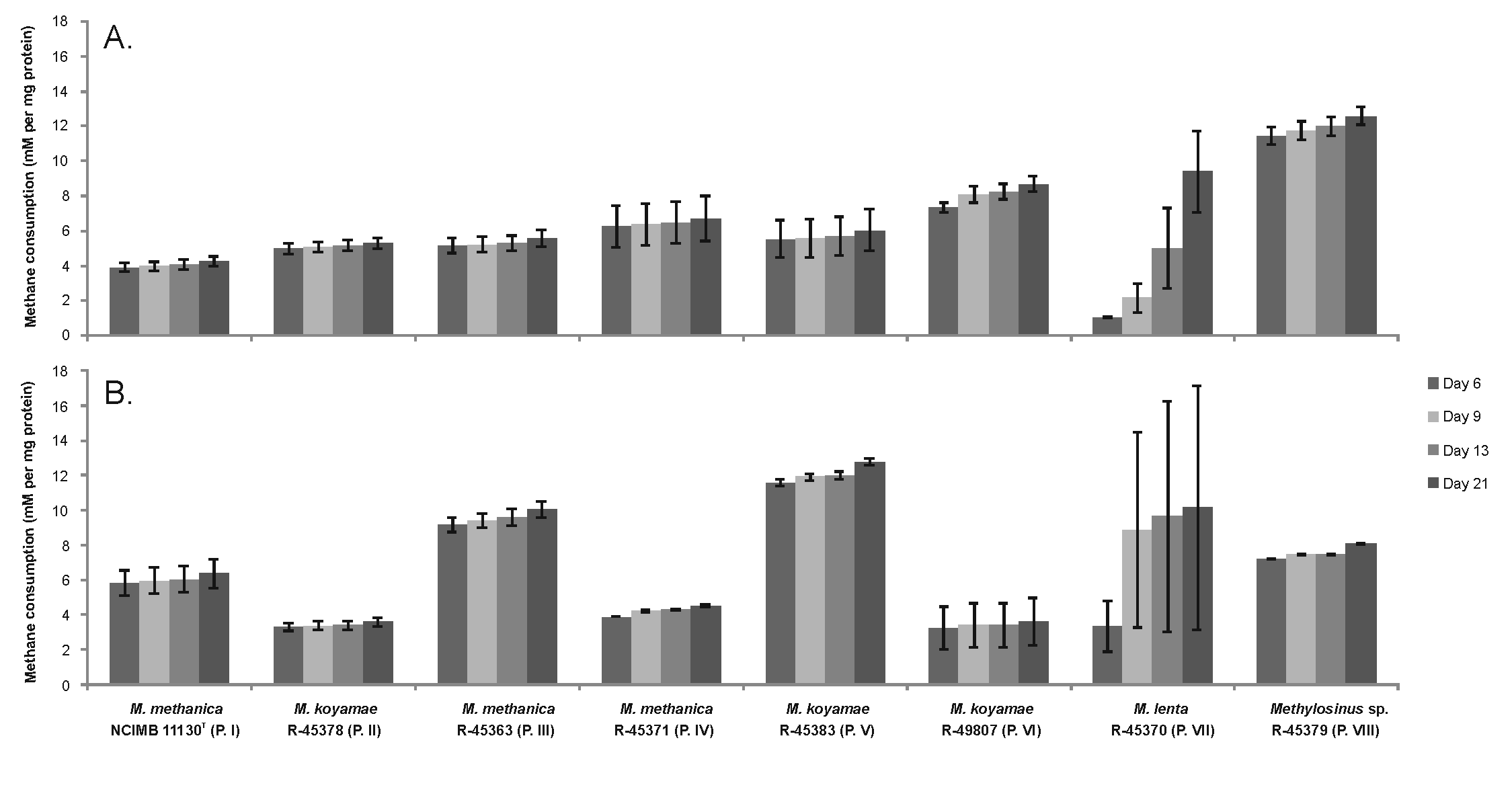

Supplement: Additional file 6: Figure S2 — Methane consumption in late exponential to stationary phase in dNMS (A) or dAMS (B) per strain (n = 2). Assigned phenotypes are indicated between brackets. Error bars are high for R-45370 because of slow growth of one replicate. [file 1471-2180-14-83-S6.tiff]

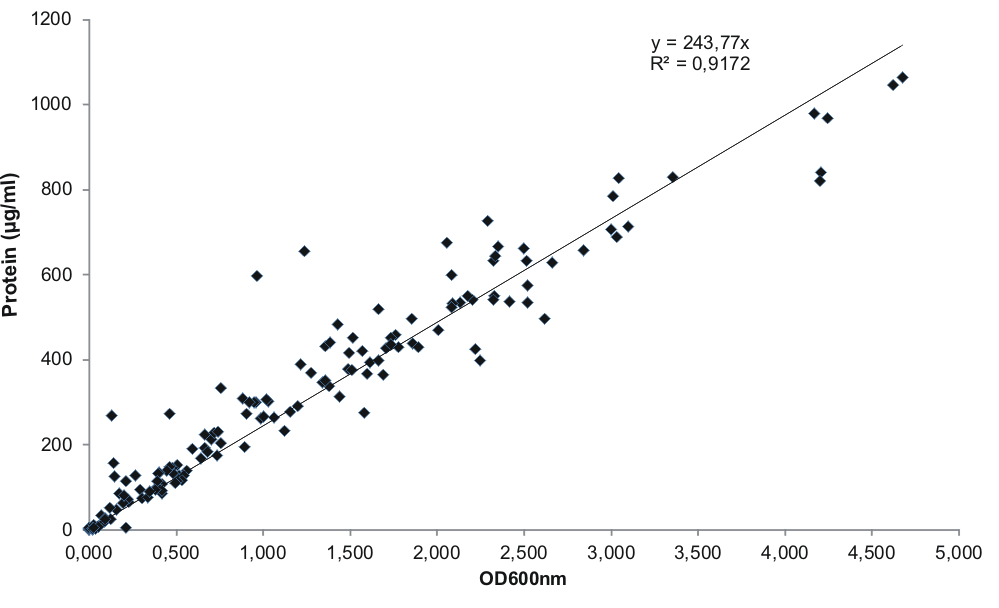

Supplement: Additional file 7: Figure S3 — Correlation between growth (OD600nm) and protein content. Data was taken from [45]. A correlation factor was extracted for conversion of growth to protein content. [file 1471-2180-14-83-S7.tiff]
