# Supplementary material for: Niche differentiation in nitrogen metabolism among methanotrophs within an operational taxonomic unit
Source: BMC Microbiol. 2014 Apr 4;14:83. doi: 10.1186/1471-2180-14-83 (PMC3997834; doi:10.1186/1471-2180-14-83)
Supplement: Additional file 3: Table S1 — Estimates of Evolutionary Divergence between 16S rRNA gene sequences. The number of base substitutions per site between 16S rRNA gene sequences are shown. Pairwise distance analyses were conducted based on the alignment used in Additional file 4: Figure S4. Evolutionary analyses were conducted in MEGA5 [51]. [file 1471-2180-14-83-S3.pdf]

Methylococcaceae_bacterium_R-49797																				
Methylomonas_aurantiaca_JB103T	0,081																			
Methylomonas_fodinarum_JB13T	0,078	0,003																		
Methylomonas_koyamae_Fw12E-YT	0,072	0,020	0,020																	
Methylomonas_koyamae_R-45378	0,070	0,020	0,018	0,002																
Methylomonas_koyamae_R-45383	0,067	0,017	0,015	0,005	0,003															
Methylomonas_koyamae_R-49799	0,072	0,020	0,020	0,000	0,002	0,005														
Methylomonas_koyamae_R-49807	0,072	0,020	0,020	0,000	0,002	0,005	0,000													
Methylomonas_lenta_R-45370	0,065	0,022	0,020	0,010	0,008	0,007	0,010	0,010												
Methylomonas_lenta_R-45377T	0,065	0,022	0,020	0,010	0,008	0,007	0,010	0,010	0,000											
Methylomonas_methanica_R-45362	0,072	0,022	0,022	0,007	0,008	0,008	0,007	0,007	0,010	0,010										
Methylomonas_methanica_R-45363	0,072	0,022	0,022	0,007	0,008	0,008	0,007	0,007	0,010	0,010	0,000									
Methylomonas_methanica_R-45364	0,072	0,022	0,022	0,007	0,008	0,008	0,007	0,007	0,010	0,010	0,000	0,000								
Methylomonas_methanica_R-45371	0,072	0,022	0,022	0,007	0,008	0,008	0,007	0,007	0,010	0,010	0,000	0,000	0,000							
Methylomonas_methanica_R-45372	0,072	0,022	0,022	0,007	0,008	0,008	0,007	0,007	0,010	0,010	0,000	0,000	0,000	0,000						
Methylomonas_methanica_R-45374	0,072	0,022	0,022	0,007	0,008	0,008	0,007	0,007	0,010	0,010	0,000	0,000	0,000	0,000	0,000					
Methylomonas_methanica_S1T	0,070	0,027	0,027	0,012	0,014	0,014	0,012	0,012	0,016	0,016	0,006	0,006	0,006	0,006	0,006	0,006				
Methylomonas_paludis_MG30T	0,065	0,024	0,023	0,011	0,009	0,009	0,011	0,011	0,006	0,006	0,014	0,014	0,014	0,014	0,014	0,014	0,020			
Methylomonas_rubra_ACM_3303	0,070	0,024	0,024	0,012	0,014	0,011	0,012	0,012	0,007	0,007	0,012	0,012	0,012	0,012	0,012	0,012	0,018	0,010		
Methylomonas_scandinavica_SR5T	0,070	0,023	0,023	0,012	0,013	0,010	0,012	0,012	0,007	0,007	0,012	0,012	0,012	0,012	0,012	0,012	0,017	0,009	0,004	
Methylosinus_sp._R-45379	0,139	0,159	0,157	0,155	0,155	0,153	0,155	0,155	0,151	0,151	0,157	0,157	0,157	0,157	0,157	0,157	0,154	0,151	0,155	0,154
