# Supplementary material for: Niche differentiation in nitrogen metabolism among methanotrophs within an operational taxonomic unit
Source: BMC Microbiol. 2014 Apr 4;14:83. doi: 10.1186/1471-2180-14-83 (PMC3997834; doi:10.1186/1471-2180-14-83)
Supplement: Additional file 5: Table S2 — Estimates of Evolutionary Divergence between pmoA gene sequences. The number of base substitutions per site between pmoA gene sequences are shown. Pairwise distance analyses were conducted based on the alignment used in Additional file 4: Figure S5. Evolutionary analyses were conducted in MEGA5 [51]. [file 1471-2180-14-83-S5.pdf]

Methylomonas_methanica_R-45362																
Methylomonas_methanica_R-45363	0,063															
Methylomonas_methanica_R-45364	0,069	0,047														
Methylomonas_methanica_R-45371	0,063	0,000	0,047													
Methylomonas_methanica_R-45372	0,063	0,000	0,047	0,000												
Methylomonas_methanica_R-45374	0,003	0,066	0,066	0,066	0,066											
Methylomonas_methanica_S1T	0,095	0,095	0,104	0,095	0,095	0,092										
Methylomonas_lenta_R-45377T	0,098	0,095	0,044	0,095	0,095	0,095	0,127									
Methylomonas_lenta_R-45370	0,087	0,081	0,064	0,081	0,081	0,084	0,107	0,036								
Methylomonas_koyamae_R-45383	0,130	0,125	0,140	0,125	0,125	0,133	0,149	0,166	0,144							
Methylomonas_koyamae_R-45378	0,102	0,103	0,115	0,103	0,103	0,105	0,103	0,145	0,139	0,145						
Methylomonas_koyamae_NCIMB_14606T	0,066	0,089	0,089	0,089	0,089	0,063	0,080	0,118	0,107	0,127	0,047					
Methylomonas_koyamae_R-49799	0,086	0,097	0,094	0,097	0,097	0,083	0,103	0,124	0,122	0,142	0,055	0,034				
Methylomonas_koyamae_R-49807	0,086	0,097	0,094	0,097	0,097	0,083	0,103	0,124	0,122	0,142	0,055	0,034	0,000			
Methylomonas_paludis_MG30T_(HE801217)	0,161	0,146	0,149	0,146	0,146	0,158	0,147	0,158	0,133	0,184	0,166	0,159	0,162	0,162		
Methylococcaceae_bacterium_R-49797	0,411	0,404	0,414	0,404	0,404	0,410	0,391	0,420	0,415	0,409	0,391	0,395	0,388	0,388	0,365	
Methylosinus_sp._R-45379	0,500	0,453	0,468	0,453	0,453	0,499	0,479	0,498	0,483	0,444	0,445	0,462	0,466	0,466	0,460	0,379
